# Immune Responses to Influenza Virus and Its Correlation to Age and Inherited Factors

**DOI:** 10.3389/fmicb.2016.01841

**Published:** 2016-11-22

**Authors:** Azadeh Bahadoran, Sau H. Lee, Seok M. Wang, Rishya Manikam, Jayakumar Rajarajeswaran, Chandramathi S. Raju, Shamala D. Sekaran

**Affiliations:** ^1^Department of Medical Microbiology, Faculty of Medicine, University of MalayaKuala Lumpur, Malaysia; ^2^Department of Biochemistry and Molecular Medicine, Faculty of Medicine, MARA University of TechnologySelangor, Malaysia; ^3^Department of Trauma and Emergency Medicine, University Malaya Medical CentreKuala Lumpur, Malaysia; ^4^Department of Molecular Medicine, Faculty of Medicine, University of MalayaKuala Lumpur, Malaysia

**Keywords:** influenza virus, immune responses, antibodies, T cells, age associated factors, inherited factors

## Abstract

Influenza viruses belong to the family Orthomyxoviridae of enveloped viruses and are an important cause of respiratory infections worldwide. The influenza virus is able to infect a wide variety species as diverse as poultry, marine, pigs, horses, and humans. Upon infection with influenza virus the innate immunity plays a critical role in efficient and rapid control of viral infections as well as in adaptive immunity initiation. The humoral immune system produces antibodies against different influenza antigens, of which the HA-specific antibody is the most important for neutralization of the virus and thus prevention of illness. Cell mediated immunity including CD4^+^ helper T cells and CD8^+^ cytotoxic T cells are the other arms of adaptive immunity induced upon influenza virus infection. The complex inherited factors and age related changes are associated with the host immune responses. Here, we review the different components of immune responses against influenza virus. Additionally, the correlation of the immune response to age and inherited factors has been discussed. These determinations lead to a better understanding of the limitations of immune responses for developing improved vaccines to control influenza virus infection.

## Introduction

Influenza virus is a major pathogen that represents an ongoing health threat to several species as diverse as poultry, swine, and mammals including humans, generally via respiratory morbidity and mortality (Eyer and Hruska, 2013). Influenza virus is a member of the *Orthomyxoviridae* family with an enveloped, negative sense-single stranded RNA (Zhang et al., 2013). They can be classified into three types: A, B, and C. The influenza A virion genome consists of eight RNA segments that are varying in sizes, with coding ability of 11 proteins, including Hemagglutinin (HA), Neuraminidase (NA), Matrix proteins (M1 and M2), Polymerase basic protein (PB1, PB2 and PA), Nucleocapsid protein (NP), PB1-F2 and non-structural proteins (NS1 and NS2; Oh and Hurt, 2014). HA functions as a mediator for virus entry into the cell by membrane fusion activity and receptor binding. Meanwhile, NA mediates the progeny virions release by viral receptor enzymatic cleavage. Integral membrane protein, M2, is a multi-functional, proton-selective, ion channel which has roles in both virus entry as well as in virus assembly and budding. The matrix protein (M1) plays an important role in the virion structure and also as a mediator for the ribonucleoprotein (RNP) core and the viral lipid membrane. PA, PB1, PB2 and NP make up the RNP core which plays a critical role in mediating the packaging and binding of the viral genome. NS1, NS2, nuclear export protein (NEP) and PB1-F2 are the three other proteins which are expressed during replication of the virus and are not merged to the mature virion (Coleman, 2007; Zhang et al., 2013). It has been investigated that NS1 protein acts as a immunosuppressor by inhibiting type I IFN release and attenuates the capacity of dendritic cells (DCs) to induce T cell responses and maturation resulting in inhibition of innate and adaptive immunity, respectively (Fernandez-Sesma et al., 2006). Four envelope proteins including HA, NA, NB and BM2 form the organization of influenza B virion. BM2 protein is similar to M2 of influenza A virus while the hemagglutinin-esterase-fusion (HEF) protein is a major surface glycoprotein of the influenza C viruses. The functionality of this protein corresponds to the HA and NA of influenza A and B viruses as well as the minor envelope protein, CM2 (Lamb and Krug, 2001).

### Replication Cycle

Influenza virus replication initiates with virus entry into the host cell via a process of receptor mediated endocytosis. The virus attaches to sialic acid-containing receptors via the HA molecule. Two main types of interaction between galactose (Gal) and sialyloligosaccharides (SAs) are SA-α2, 3-Gal and SA-α2, 6-Gal. Normally HA proteins of avian influenza virus (AIV) bind to the SA-α2 and 3-Gal preferentially while a higher affinity for SA-α2 and 6-Gal linkage is observed for HA proteins of human influenza virus. The viral entrance into the cell is through the endocytic pathway. The low pH of endosome causes a change in the HA protein conformation leading to exposure of a hydrophobic fusion peptide. After internalization and fusion of the vesicle with the endosome, the virus enters into the cytoplasm and the released viral RNP complexes are transported into the nucleus. In the nucleus, viral mRNA and complementary RNA (cRNA) will be synthesized from the vRNPs templates. The synthesized mRNAs will be exported into cytoplasm for translation of viral proteins. These newly synthesized proteins are transported to the nucleus for final assembly of vRNP. cRNAs are then used as template for synthesis of more negative sense viral RNA for packaging into progeny virions and amplification of mRNA synthesis. Finally, viral nucleocapsides are assembled in the nucleus before being transported back into the cytoplasm and subsequently form buds at the plasma membrane followed by release of the new viral particles (Julkunen et al., 2001; Coleman, 2007).

## Immune Responses of Influenza Infection

### Innate Immunity

#### Pathogen Recognition Receptor of Influenza Virus

**FIGURE 1:**
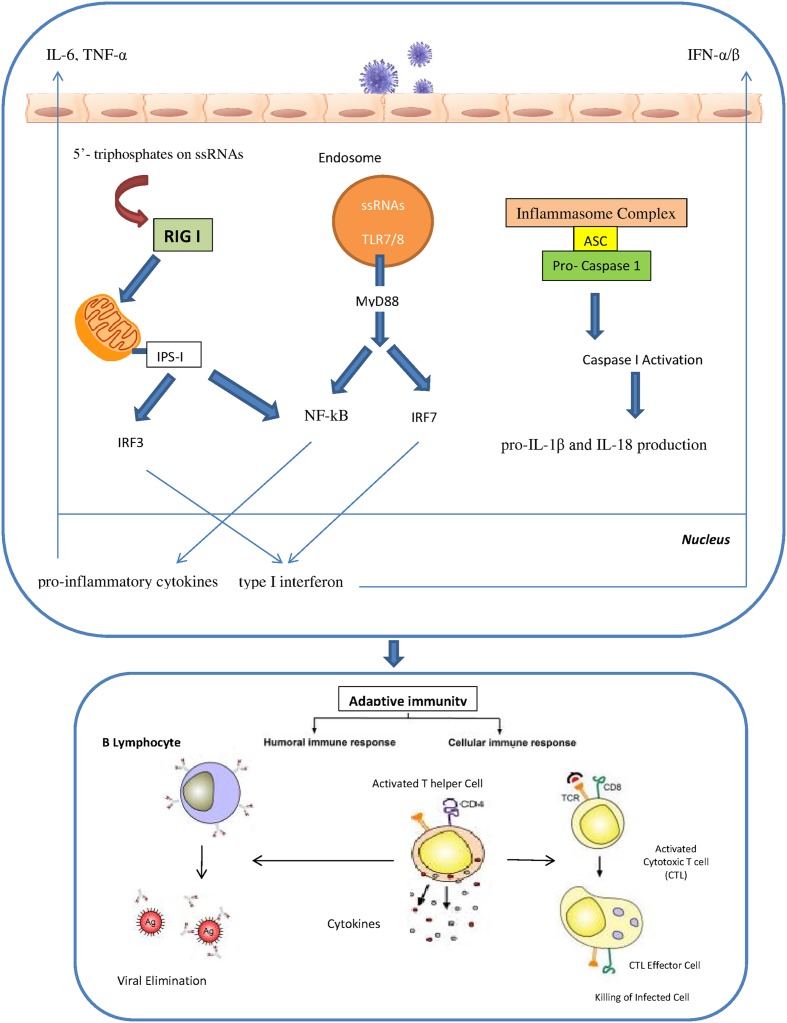
**The innate and adaptive immunity against influenza virus**.

The innate immunity plays a critical role in efficient and rapid limitation of viral infections as well as for adaptive immunity initiation. Different pathogen recognition receptors (PRRs) in the cells of the innate immune system are utilized to recognize the influenza A virus. There are three different PRRs to sense influenza A virus, which includes the retinoic acid inducible gene I (RIG-I), the Toll-like receptors TLR3, TLR7 and TLR8 and nucleotide binding oligomerization domain (NOD)-like receptors (NLRs; Pang and Iwasaki, 2011; Tripathi et al., 2013). RIG-I is a cytosolic sensor that recognizes the influenza virus through detection of 5′- triphosphates on single stranded RNAs. In specialized cells like plasmacytoid dendritic cells (pDCs), single stranded viral RNA is exposed by viral capsid degradation in the acidified endosomes for detection by TLR7. The production of pro-inflammatory cytokines and type I interferons are induced through the RIG I and TLR7 pathways. IRF7 (interferon regulatory factor 7) and NF-kB are activated by TLR7 signals which are mediated by MyD88 protein as an adaptor protein. In contrast, RIG I signals can activate the IRF3 and NF-kB through the IPS I protein which is located in the mitochondria. Activated IRF3 and IRF7 are then translocated into the nucleus to induce type I interferon production. Meanwhile, NF-kB activates the pro-inflammatory cytokines such as IL-6, TNF-α and IL-1 β. Human monocytes and macrophages express TLR8 and also produce IL-12. However, the relevance of TLR8 in influenza virus infection is still unidentified. Recently, several studies have revealed a significant role of cytoplasmic complexes called inflammasomes in influenza virus detection. The cytoplasmic inflammasome complex including the NLR-subset (namely NLRP1, NLRP3 and NLRC4) is able to activate caspase-1 resulting in the pro-IL-1β and IL-18 production (Loo et al., 2008; Rehwinkel et al., 2010; Pang and Iwasaki, 2011; Latz et al., 2013; Pothlichet et al., 2013; Tripathi et al., 2013; Iwasaki and Pillai, 2014; Abderrazak et al., 2015) (**Figure 1**). IFN-α/β, IFN-γ and IFN-λ are the results of activation of all these pathways that led to the induction of the antiviral response and the activation of neutrophils, recruitment of macrophages and maturation of DCs (Sanders et al., 2011). Activation of IFN-α/β as type I IFNs results in antiviral signaling cascades that involves phosphorylation of tyrosine kinase 2 (Tyk2) and Janus kinase 1 (Jak1), followed by phosphorylation of signal transducer and activators of transcription (STAT) 1 and STAT2. Finally IFN-stimulated gene factor-3 transcription factor complex (ISGF3) is formed through the combination of phosphorylated STAT1 and 2 with IRF9, resulting in the establishment of an antiviral state in the cell. IFN-γ is the major type II IFN that establishes an efficient adaptive and memory cytotoxic T cell response against the influenza virus infection (Bot et al., 1998; Turner et al., 2007). IFN-λ, a type III IFN, is responsible for control of influenza A infection in the lung (Mordstein et al., 2008). One study showed that after influenza A virus infection pDCs produced higher concentrations of IFN-λ than monocyte-derived DCs thus implying that pDCs are the primary cells in IFN-λ production (Ank et al., 2006).

#### Macrophages, Natural Killer Cells and Dendritic Cells

The initial phase of the influenza virus infection activates alveolar macrophages and monocytes which results in a pro-inflammatory cytokine response involving TNF-α and IL-6. Alveolar macrophages have beneficial effects in limiting viral spread either by phagocyte-mediated opsonophagocytosis of influenza virus particles and phagocytosis of apoptotic infected cells or by regulating the adaptive immune response (Huber et al., 2001; Tumpey et al., 2005; Hashimoto et al., 2007; Kim et al., 2008). Natural killer (NK) cells as cytotoxic lymphocytes are critical for elimination of influenza virus infection through two mechanisms. First, they bind to HA proteins through sialylated NKp44 and NKp46 receptors. Second, NK cells are able to attach to the Fc portion of antibodies bound to the influenza virus-infected cells through their CD16 receptor to mediate lysis of these cells (Guo et al., 2011; van de Sandt et al., 2012). DCs as professional antigen presenting cells are characterized as a critical mediator between the innate and the adaptive immune system. The adaptive immune response is initiated when DCs present viral antigens to the naïve and memory T lymphocytes. DCs constantly inspect the lungs for foreign materials or invading pathogens. During an influenza virus infection, the antigens are acquired by the DCs through two different mechanisms. The first mechanism is by direct infection of DCs, whereby the viral proteins are degraded into small peptides by proteasomes in the cytosol, transported to the endoplasmic reticulum (ER) and subsequently loaded to major histocompatibility complex (MHC) class I molecules. The MHC class I/peptide complexes are recognized by the virus-specific CD8+ cytotoxic T cells (CTLs). Phagocytosis of virus particles or apoptotic epithelial cells is the second mechanism of antigen acquisition by DCs. Degraded viral proteins are bound to the MHC class II molecules and these complexes presented on the cell surface can be recognized by CD4+ T helper cells, which would then lead to B cells proliferation and maturation into antibody-producing plasma cells. DCs are also able to present epitopes to CD8+ T cells via this route of antigen acquisition which is known as cross-presentation (van de Sandt et al., 2012).

### Adaptive Immunity

#### Humoral Immunity

The humoral immune system produces antibodies against different influenza antigens. Understanding host antibody response is crucial to predict disease severity and for vaccine development. The HA-specific antibody is the most important for virus neutralization to prevent illness through binding to the trimeric globular head of HA, thus inhibiting virus attachment to the host cells. Additionally, HA-specific antibodies can bind to the infected Fc receptor-expressing cells to facilitate phagocytosis of virus particles (de Jong et al., 2000). There are also antibodies directed against the highly conserved stem region of the HA, with the ability to neutralize different subtypes of influenza viruses although the titers are low (Ekiert et al., 2009, 2011; Sui et al., 2009). Such antibodies would be useful in stimulating an immune response against all HA types, however, this has yet to be proven. Antibodies against NA limit virus spread by inhibiting enzymatic activity and additionally by facilitating antibody-dependent cell-mediated cytotoxicity (ADCC). M2-specific antibodies are produced to a limited extent after natural infection as this protein itself is present at low concentrations in the infected cells. Besides, NP-specific antibodies may also contribute during influenza virus infection. Although the mechanism of protection remains to be clarified, these antibodies are able to trigger complement mediated cell lysis of infected cells (Carragher et al., 2008; LaMere et al., 2011). However, both antigenic drift and shift of the surface antigens could reduce the effectiveness of antibody binding to the HA and NA, hence leading to renewed susceptibility to infection. Nevertheless, heterotypic antibody can convey substantial immunity depending upon the extent of cross-reactivity for infecting virus antigens. While serum anti-HA antibody is the major need for optimal immunity to influenza, a full complement of immune modalities is desirable to ensure maximum immunity (Couch, 2002).

The main entrance for many pathogens such as influenza is the mucosal tissues. Thus, IgA and to some extent, IgM, may act as the main antibodies in the mucosal tissues by neutralizing the mucosal pathogens and subsequently to prevent pathogen entry and virus replication. Neutralizing antibodies, primarily of the IgA isotype, act especially against the HA and NA of influenza virus. Induction of primary response occurs in organized lymphoid tissues while secondary responses may occur in the periphery. During primary responses, IgM antibody is initially dominant whereas IgG antibody is dominant during secondary responses (Cox et al., 2004; Kreijtz et al., 2011; van de Sandt et al., 2012). It was shown that a higher IgM level could be associated with a quicker viral clearance and an early good IgM response is usually necessary for a subsequent good IgG antibody response. This indicates that there may exist an innate linkage between early influenza A-specific IgM response and subsequent IgG antibody production (Qiu et al., 2011). Generally the half-life of antibodies is short but Ab titers can last a lifetime due to a subset of the Ab-secreting cells (ASCs) which is long-lived. It has been shown in the study that transmembrane activator, calcium modulator, cyclophilin ligand interactor (TACI) cytokines, B lymphocyte stimulator (BLyS) and proliferation-inducing ligand (APRIL) have critical roles in producing optimum humoral immunity and most important in providing protection against secondary viral infection. Targeting TACI on both innate cells and B cells for greater antiviral ASC survival can lead to enhanced Ab titer maintenance and protection (Wolf et al., 2011).

#### Cellular Immunity

CD4+ and CD8+ T cells are induced upon infection with influenza virus. Activation of virus specific CD4+ helper T cells, both Th1 and Th2 type cells, recognize virus-derived MHC class II-associated peptides on antigen presenting cells, followed by expression of co-stimulatory molecules (Tamura and Kurata, 2004). Additionally, T helper 17 (Th17) and regulatory T cells (Tregs) that control the cellular immune response against influenza virus infection have been identified. Th17 cells improve T helper reactions by production of IL-6 which prevents Tregs function. Tregs control both the CD8+ T cell and the T helper cell responses after infection. Besides, Tregs do not have any effect on the B cell response but they are able to suppress the T helper response (Surls et al., 2010; Campbell and Koch, 2011). Furthermore, IL-35 secreted by Tregs, acts as suppressor to inflammatory responses. It has also been shown that IL-35 is upregulated during secondary pneumococcal pneumonia following influenza infection (Chen et al., 2016).

Some cytolytic activity of CD4+ T cells is displayed in infected cells. However, T helper (Th) cells are the most significant phenotype of these cells. Th cells are divided into two subsets including Th1 and Th2 cells based on their different cytokine expression profile. Th1 cells produce IFN-γ and IL-2 and are involved predominantly in cellular immune response whereas Th2 cells induce IL-4 and IL-13 production and are shown to stimulate B cell responses (Kreijtz et al., 2011). The study on genetically modified mice with the IFN-γ gene disruption showed enhanced Th2 cytokines induction and influenza-specific IgG1 antibody production. However, cell mediated immune responses in these mice were similar to the wild type control. Remarkably, *in vitro* re-stimulation of CD4 cell clones which were isolated from mice with deficient IFN-γ were able to protect against lethal challenge with influenza virus through a cytolytic mechanism. Furthermore, CD4 cells showed the ability to lyse Sendai or LCMV infection in virally infected class-I-deficient mice indicating the capability of CD4 cells to become killers themselves for compensation of deficiency in IFN-γ or CD8 cells (Brown et al., 2004). The ability of CD4+ T cells to prompt antiviral B cell responses is the most significant contribution of these cells, which lead to class-switching of antibody, affinity maturation, and generation of long-lived plasma cell. Distinct CD4+ T cell subset is considered for this effector activity named the T follicular helper (Tfh) cell. Since the Tfh cell responses can have effective implications for immunological memory and vaccination, it is an area of extreme interest in experimental influenza infection (Kim et al., 2011).

Upon influenza virus infection, viral epitopes associated with MHC class I molecules activates the naïve CD8+ T cells in the draining lymph nodes, which subsequently differentiates into cytotoxic T lymphocytes (CTLs; Kreijtz et al., 2011; van de Sandt et al., 2012). Activation of these cells leads to migration to the infection site where they detect influenza virus-infected cells and eliminate them via lytic activity and hence inhibit the virus progeny production. The lytic activity is facilitated by the perforin and granzymes (e.g., GrA and GrB) secretion (Metkar et al., 2008). The infected cell membrane is permeabilized by perforin to assist the entrance of granzymes into the cells and finally apoptosis induction. Furthermore, GrA shows non-cytotoxic activities focusing on the prevention of virus replication via cleavage of viral proteins and host cell proteins that are involved in protein synthesis (Andrade, 2010; Domselaar and Bovenschen, 2011). CTLs also have the capability to prompt apoptosis of infected cells via Fas/FasL interactions. Moreover, they generate cytokines that increase antigen-presentation by inducing MHC expression. Post-infection virus-specific CTL which are developed and preserved under the regulation of T cell-produced IL-17 and DCs is located in the blood circulation, the lymphoid organs and also at the site of infection (GeurtsvanKessel et al., 2009; Rangel-Moreno et al., 2011). These memory CTL cells are able to activate upon secondary influenza virus infection. The co-stimulation that they received during their initial differentiation phase affects their reactivity and affinity during a secondary infection (van Gisbergen et al., 2011). Human CTL produced by influenza virus infection are primarily directed against NP, M1 and PA proteins. These proteins are highly conserved and therefore the CTL response is cross reactive to a high degree even between different subtypes of influenza A viruses (Kreijtz et al., 2011).

## Influence of Age and Inherited Factors on Immune Responses to Influenza

Toll-like receptors are one the most important PRRs involved in influenza virus recognition. There are strong evidences showing correlation between age-associated changes and expression and function of TLR. In mouse models, lower expression of TLR2, TLR3, TLR4, TLR5, TLR6, TLR7, TLR8, and TLR9 was demonstrated in macrophages of aged mice, which resulted in the reduction of proinflammatory cytokines such as interleukin (IL)-6 and tumor necrosis factor (TNF) α upon stimulus with specified ligands for TLRs (Renshaw et al., 2002; Boehmer et al., 2005). Additionally, reduced expressions of TLR1, TLR3 TLR8 and TLR7 have been demonstrated in human DCs as well as lower production levels of IL-6, TNFα, IL-12 and interferon α (IFNα) upon stimulation with different TLR ligands in elderly individuals. Similarly, lower levels of TNFα upon TLR4 or TLR1/2 stimulation by monocytes and macrophages were noted in elderly individuals (Agius et al., 2009; Panda et al., 2010). pDCs from aged donors secrete reduced amounts of both IFN-1 and IFN-III after stimulation with both the TLR7 ligand CpG and live influenza virus and they also show diminished priming of CD4/CD8 T-cell immunity and antibody production (Čičin-Šain et al., 2010; Panda et al., 2010; Sridharan et al., 2011). CD80 and CD86 as costimulatory molecules expressed on antigen-presenting cells interact with CD28 and help to activate T cells (Tsukamoto et al., 2009). Investigations also showed that TLR-induced CD80 levels were significantly lower in older adults than young adults. Taken together, both cellular and humoral immunity to influenza are affected directly by a reduced TLR response in immune cells from older adults (Van Duin et al., 2007).

Dendritic cells are characterized as a crucial component of the innate immune system and also for the initiation of adaptive immunity. Antigen recognition by PRRs is accompanied by migration of DCs to regional lymph nodes followed by differentiation into mature DCs. Furthermore, some cytokines and chemokines are produced by DCs to activate and to differentiate T and B cells. Different studies on DCs from elderly individuals demonstrated a reduction in antigen capture, migration and finally T-cell activation capacity (Agrawal et al., 2007; Grolleau-Julius et al., 2008; Agius et al., 2009). Specifically, decreased upregulation of the costimulatory molecules CD86, CD80 and human leukocyte antigen (HLA) class I molecules on DCs from elderly individuals was observed after influenza stimulation. NK cells are also important in clearance of viral infection by IFN-γ production and in lysis of infected cells. One interesting study showed that the NK cell number is enhanced in healthy older adults, while in contrast a reduced number of NK cells was observed in older adults with a weakened health type leading to an assumption that the activity of NK cells is related to health status of older adults (Myśliwska et al., 2004; Zhang et al., 2007). Moreover, age is associated with alterations in the function and phenotype of NK cells. In healthy elderly individuals, the cytotoxic activity of NK cells is decreased at the single cell level but as numbers of NK cells increased, total NK cell mediated cytotoxicity is preserved (DelaRosa et al., 2006). In contrast, lower NK cell-mediated cytotoxicity has been investigated in frail individuals and elderly with chronic diseases (Bruunsgaard et al., 2001). No CD16 expression and their ability to activate antibody dependent NK cell cytotoxicity are influenced by age (DelaRosa et al., 2006). Regarding the phenotypes, aging causes to decrease the expression of some cytotoxicity activating receptors such as DNAM-1, NKp30 and NKp46 (Bruunsgaard et al., 2001). The effect of age on the inhibitory receptors expression of NK cells such as KIR receptor or CD94/NKG2A is very controversial. Some studies did not show significant differences in the expression of CD94/NKG2A and KIR in elderly compared with young individuals (Garff-Tavernier et al., 2010) while the others found an enhancement in the KIR expression and a reduction expression of CD94/NKG2A receptor in elderly (Lutz et al., 2005).

Several data showed that aging significantly affects humoral immune responses through noticeable effects on generation and function of B cells. It has been demonstrated that production of high affinity antibodies is diminished in elderly population because of some deficiencies in somatic hypermutation and isotype switching (Frasca et al., 2005). Moreover, decreased diversity of antibody reactions was observed with aging due to an ongoing reduction in naïve B cells and hence an observed decrease in effector B cells (Allman and Miller, 2005). Additionally, an impaired ability of bone marrow cells to support survival of antibody-producing plasma B cells was observed in older mice (Han et al., 2003).

**FIGURE 2:**
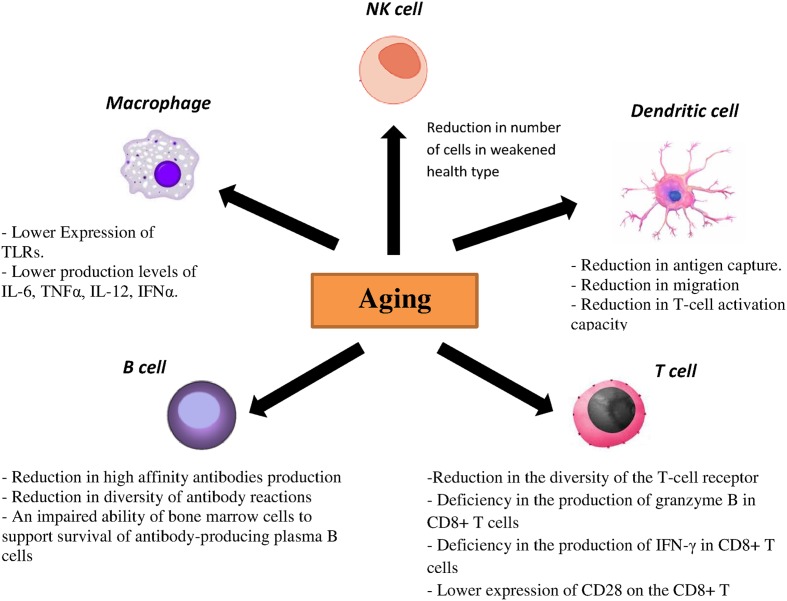
**Effect of aging on the immune responses**.

Within the T-cell compartment age-related changes are also evident. As involution of the thymus starts early in life, the output of naïve T cells reduces considerably with age, resulting in decreased numbers of naïve T cells in the periphery in elderly individuals. Lifespan and homeostatic turnover of naïve T cells will increase in order to compensate the decreased peripheral T cells which significantly cause a reduction in the diversity of the T-cell receptor (TCR) repertoire. Furthermore, a decreased TCR repertoire has been related to an inadequate vaccination response and reduced immunity against influenza virus (Čičin-Šain et al., 2007; Kilpatrick et al., 2008; Ahmed et al., 2009; Tsukamoto et al., 2009). Moreover, a deficiency in the production of granzyme B in CD8+ T cells as well as IFN-γ has been demonstrated in vaccinated older adults (Lee et al., 2011; Zhou and McElhaney, 2011). Another molecule, CD28, is a critical component that differentiates naïve T cells after antigen exposure. It was shown that the expression of this molecule on the CD8+ T cells decreases with age leading to a poor immune response to influenza vaccine (Goronzy et al., 2001). Aging also causes the accumulation of highly differentiated effector-memory CD8 T cells with characteristics of replicative senescence. In this case the capacity to proliferate decreases, telomeres are shortened, telomerase activity is lost and CD28 expression is reduced (Pera et al., 2015). The effect of aging on the immune responses has been summarized in **Figure 2**.

It is generally believed that multiple defects in the immune responses including diminished anti-hemagglutinin (HA) antibody titers as well as diminished specificity of these antibodies causes a reduction in the efficacy of influenza vaccine in the elderly (Sasaki et al., 2011). These reduced responses are linked to a reduced heterogeneity in the B cell pool and reduced production of B cell precursors in the bone marrow, as mentioned earlier. As infectious disease in the elderly imposes high economic and social costs, most developed countries provide a comprehensive program to vaccinate the individuals over the age of 65 (Trzonkowski et al., 2009). Advances in our understanding about alterations in immune system in the elderly as well as exploiting those alterations to induce more robust and long-lasting reactions to vaccine contents may help to improve the current vaccination for the elderly. Moreover, various strategies are used to induce greater antigen-specific antibody responses and to maximize cross-protective immunogenicity including adjuvanted vaccines, alternate routes of immunization, universal vaccine strategies and high-dose vaccines (Haq and McElhaney, 2014).

Considering the different behavior of immune responses in elderly individuals, it is worth suggesting more investigations into the important protective factors such as specific and non-specific antibodies against influenza virus in the elderly. Additionally, correlation between age dependent comorbidities such as chronic respiratory conditions, metabolic disorders, cardiovascular, hepatic, renal and hematological and influenza virus infection, and its effect on the immune response needs to be investigated, as these underlying conditions may have an impact on the quality of the immune response to be generated. Besides, the need to study the potential of daily routines such as nutrition and exercising for increasing the immune responses in elderly people is also required.

The function of environmental agents in immune aging remains incompletely understood. For example, latent cytomegalovirus (CMV) infection has been associated with an immune risk profile in the elderly humans. Mouse CMV infection showed selected T-cell subset changes associated with immune aging, specifically the enhancement of relative and absolute counts of CD8 T-cells in the blood, with a decreased representation of the naïve and the increased representation of the effector memory blood CD8 T-cells. Additionally, weaker CD8 responses to superinfection with influenza were observed in mouse CMV infection, even 16 months following CMV infection (Cicin-Sain et al., 2012). It also has been documented that human CMV has negative effect on the *in vivo* and *in vitro* B cell responses to the seasonal influenza vaccine (Sansoni et al., 2014). Conversely, other studies showed that acute and early latent MCMV infection resulted in improved control of influenza virus infection. The lungs of influenza-infected animals have been filtered by MCMV-specific CD8+ T cells several days prior to the influenza-specific CD8+ T cell response. This ability is due to producing significant amounts of the cytokines IFN-γ and TNF-α (Barton et al., 2007; Furman et al., 2015). Although multiple studies suggest that persistent CMV infection is associated with accelerated aging of the immune system and with several age-related diseases, however, how CMV infection is implicated in immunosenescence and in age-related diseases remains controversial (Sansoni et al., 2014).

It is also important to mention that besides age-related changes, the immune system shows significant sex-specific differences. It is well established that the aging process affects sexual dimorphism regarding immunocompetence and disease susceptibility. This influences on the etiopathology of infectious diseases like influenza virus as main causes of morbidity and mortality in older people (Giefing-Kröll et al., 2015). Particularly, men exhibited to be more concerned by seasonal influenza, whereas premenopausal women surrender more often to pandemic strains. Moreover, it has been investigated that, enhanced stimulation of pro-inflammatory cytokines and chemokines are associated with higher morbidity and mortality in infected-influenza virus women (Klein et al., 2012; Quandelacy et al., 2014). However, stronger humoral responses were observed in vaccination against influenza in women. These results suggest that conclusively during aging, interventions, which distinctively consider the changing level of individual hormones, may provide potent options in maintaining optimal immune functions (Giefing-Kröll et al., 2015).

Based on some evidences showing an inherited susceptibility to the influenza virus infection and death, genetic associations with the immune response and influenza infection is also intensely suggested (Albright et al., 2008). Heat shock protein gene, heme oxygenase-1 (*HO-1*) has been identified as an effective gene in immune responses to influenza virus infection. Several studies showed the relationship between the increased levels of *HO-1* gene and immune responses against influenza infection. Furthermore, decreased antibody production in response to influenza vaccination was observed in aged HO-1-deficient mice (Cummins et al., 2012). Critically, the strong association between impaired influenza vaccine responses and polymorphisms in the *HO-1* gene has been demonstrated. Hence it is essential to investigate the *HO-1* and other gene polymorphisms to improve the understanding of genetic determinants for influenza control and vaccine efficiency.

There are several studies showing the important role of host genetic polymorphisms on immunity against influenza infection. It has been revealed that HLA and other immunity-related gene polymorphisms have critical roles in induction of humoral immunity against influenza virus. HLA class I and class II molecules are responsible for the presentation of antigenic epitopes to CD8^+^ and CD4^+^ T cells, respectively, which consequently induces T-cell populations with distinct functions. The correlation between various HLA class I and class II alleles and the immune response to influenza vaccination have been reported (Marchant et al., 2006). Some studies on TLR4 as a critical component of innate immunity in the recognition of lipopolysaccharide showed that the TLR4 gene polymorphisms is important in the immune responses against respiratory syncytial virus and as a consequence, were susceptible to severe RSV disease (Marchant et al., 2006). Additionally, IFN-β generation to control the influenza infection is influenced by interaction with the *RIG-1* gene. Influenza virus NS1 protein is able to prevent the *RIG-1* pathway by interaction with *RIG-1*, resulting in inhibition of IFN-β production (Marchant et al., 2006). Significant correlations has been demonstrated between influenza H1-specific HAI antibody levels and single nucleotide polymorphisms in cytokine genes (IFN-g, IL12A, IL12B, IL18 and IL6), and cytokine receptor genes (IFNAR2, IL1R, TNFRSF1A, IL4R, IL2RG, IL12RB and IL10RB; Gelder et al., 2002; Poland et al., 2008; Cummins et al., 2012). The polymorphism of the immunoglobulin heavy chain variable region (IGHV) exhibits the diversity of the humoral immune system. The role of *IGHV1-69* germline gene to generate the neutralizing antibodies against stem region of HA of influenza virus and generally to regulate B cell function and antibody repertoire expression has been investigated (Avnir et al., 2016). Also important genes involved in maturation and function of DCs, antigen presentation, and membrane trafficking and apoptosis of infected cells mediated by TCLs have been investigated (Franco et al., 2013). In addition, one study showed the upregulation of components of the interferon pathway and innate immunity including *MX1, IFITM3*, *IRF7*, *STAT2, OAS2*, *and IFI44L* in the acute phase of infection. Investigations also showed that differential expression of genes in the recovery phase of infection is disguisable from acute phase suggesting the strong association between a gene expression signature and influenza virus infection (Zhai et al., 2015). Development of new vaccines against influenza virus needs a strong understanding of genetic effects on the immune responses. Identification of interactions between different pathways of critical gene and immune responses will result in a better understanding of the host response to influenza virus and influenza vaccine antigens. The host genetic polymorphisms associations with the immune response and influenza infection have been summarized in **Table 1**.

**Table 1:** Host genetic polymorphisms associations with the immune response and influenza infection.

Host genetic polymorphisms	Function of the molecules
Heme oxygenase-1 (HO-1)	Responsible for the anti-inflammatory response to severe influenza infection
HLA class I	Responsible for the presentation of antigenic epitopes to CD8^+^
HLA class II	Responsible for the presentation of antigenic epitopes to CD4^+^ T cells
Single nucleotide polymorphisms in TLRs	Central to antiviral innate immunity
*RIG-1*	Recognition of double-stranded RNA and contribute to the antiviral state of an infected cell and involved in the influenza virus-specific production of IFN-β.
Single nucleotide polymorphisms in cytokine (IFN-g, IL12A, IL12B, IL18 and IL6)	Central to antiviral innate immunity
Single nucleotide polymorphisms in cytokine receptors (IFNAR2, IL1R, TNFRSF1A, IL4R, IL2RG, IL12RB and IL10RB)	Central to antiviral innate immunity
Immunoglobulin heavy chain variable (IGHV)	Responsible for the diversity of the humoral immune system
MX1, IFITM3, IRF7, STAT2, OAS2, and IFI44L	Components of the interferon pathway and innate immunity


It is worth to mention that influenza epidemics extremely affect elderly people with the highest rates of morbidity and mortality. The mortality rate increases dramatically with age, with the risk in people aged 80 years and above at an approximately 11-fold higher than the people aged 65–69 years. Moreover, in recent decades, about 90% of all influenza-related deaths occurred among the senior citizens, 75% aged 70 years while 55% aged over 80 years. Additionally, poor immune responses account for reduced efficacy of vaccines in people aged over 65 years. However, for occasional severe seasons, poor immune responses with surge in deaths can often be seen in children and young adults (Simonsen et al., 2009). Studies by the Centre of Disease Control (CDC) showed that flu vaccination decreased the risk of a more serious flu effect by evolution of the immune responses. For example, a recent study showed that flu vaccine decreased children’s risk by 74% during flu seasons from 2010–2012. Another study also showed that flu vaccination caused a reduction of 71% in flu-related hospitalizations among adults and 77% among adults 50 years of age and older during the 2011–2012 flu seasons (CDC, 2013). Lower rates were also observed in some vaccinated people with heart disease and diabetes (79%) and chronic lung disease (52%). The flu vaccine was also effective in preventing hospitalization of infants and pregnant women by 92% (CDC, 2013).

## General Conclusion

Despite many efforts and investigations in the field of immune responses against influenza viruses, there are still major gaps toward understanding the different factors of immune system and their viral targets. Research focused on the immune mechanisms of protection against influenza viruses and specifically the induction of CTL responses to conserved epitopes can be useful for creating novel vaccines and other involvement approaches. Several factors like age, sex, and inherited factors in immunity against influenza infection are also complex phenomena that may cause several changes in different components of the immune system. Specifically, critical characteristics of immunosenescence include: decreased number and function of DCs; alteration in number of NK cell; decreased number of naive T and B cells; reduction in the diversity of the TCR; reduction in production of high affinity antibodies and diversity of antibody reactions. A decline in immune responses including innate and adaptive immune systems in the elderly leads to more susceptibility to infection and consequently, responsiveness of vaccine is compromised, especially in frail elderly individuals. The development and identification of immunosenescence markers in patients with impaired responses to vaccination may help to decrease morbidity and mortality of infections in the aged people. Generally, to ensure protection against influenza, development of improved treatments specifically vaccines addressing the affected parts of the immune system by several factors is needed leading to develop our ability to prevent influenza virus infections in the future.

## Author Contributions

AB, SL, and SW contributed equally toward literature search and writing of innate immune responses to influenza; JR and RM contributed toward writing of humoral responses; SS and CR contributed toward writing of cellular responses; SS and RM funded salaries of the first two authors who were employed as research assistants. RM is grant holder.

## Conflict of Interest Statement

The authors declare that the research was conducted in the absence of any commercial or financial relationships that could be construed as a potential conflict of interest.

The reviewer LI and handling Editor declared their shared affiliation, and the handling Editor states that the process nevertheless met the standards of a fair and objective review.
